# Olfactory signaling of aggressive intent in male-male contests of cave crickets (*Troglophilus neglectus*; Orthoptera: Rhaphidophoridae)

**DOI:** 10.1371/journal.pone.0187512

**Published:** 2017-11-07

**Authors:** Nataša Stritih, Alenka Žunič Kosi

**Affiliations:** National Institute of Biology, Department of Organisms and Ecosystems Research, Ljubljana, Slovenia; Institute of Animal Science, CZECH REPUBLIC

## Abstract

In animal contests, communicating aggressive motivation is most often mediated by visual or acoustic signals, while chemical signals are not expected to serve such a function since they are less able to be modulated by the sender during the changing behavioral context. We describe a rare example of ephemeral olfactory signals in terrestrial animals, signals that are emitted via protrusive scent glands in male cave crickets *Troglophilus neglectus* (Orthoptera, Rhaphidophoridae) to reflect the state of the signaler’s aggression. We correlate the intensity of behaviorally expressed aggression of the individuals in dyadic contests with the frequency and extent of their gland tissue protrusion, the latter serving as an indication of the amount of released odor. We detected large amounts of odor release during brief gland protrusions, and the absence of its release during gland retraction. Males protruded the glands during and after encountering a rival, with the degree of protrusion increasing with the intensity of the signalers’ aggression. During the encounters, the degree of gland protrusion increased most strongly with the occurrence of the elevated body posture, directly preceding the attack. This degree was significantly higher in encounter winners than in losers displaying such posture, suggesting the highly important role of the released odor for contest resolution. After the encounters, glands were protruded almost exclusively by winners, apparently announcing victory. We tested for the function of the olfactory signals also directly, by preventing gland tissue protrusion in symmetric and asymmetric treatments of the contestants. Treating only the dominant individuals decreased the percentage of encounters they won by over 60%, while treating both contestants elicited a significant increase in the frequency and duration of fights. During contests, the olfactory signals of *T*. *neglectus* apparently function as a highly effective threat, which prevents maximal contest escalation and decreases the conflict-related costs.

## Introduction

In animal contests over resources, the opponents most often exchange information via signals and displays to determine the superior individual prior to engagement in a physical fight [1, 2, 3]. This communication reflects mainly two kinds of traits: the physical and physiological ability of the contestants to win a fight (the “resource holding power”; *sensu* [4]), and their motivation to fight [1, 2, 3]. Aggressive motivation, also referred to as the state of intrinsic aggressiveness [5] or willingness to fight [6], reflects an individual’s contemporary need for a resource, which depends on internal factors such as the level of hunger or reproductive state, and on the quality and importance of, as well as the available information about a resource [1, 7]. As opposed to an individual’s fighting ability, which is communicated to the opponent by an inherently honest index or by handicap signals [8, 9], information on aggressive motivation is often encoded by conventional signals that are arbitrarily linked to the signaled attribute [10, 11, 12].

Dynamic conventional signals that can vary over short periods, such as visual or acoustic, are especially suitable for conveying information about short-term changes in the motivational state or intent of the signaler [10, 13]. Such signals may generally function as challenges emitted from a distance, as general aggressive motivation signals that reflect the need for a resource once the opponent has been detected, or as offensive threats that signal a high probability of subsequent attack [1]. Threat signals often occur in the form of pre-attack postures or movements, frequently in concert with presentation of weapons [14, 15], but may also be purely conventional visual or acoustic displays (e.g. [16, 17, 18, 19]). Chemical signals are generally not supposed to signalize aggressive motivation and, particularly, the intention to attack, since they not only are difficult to modulate but also provide poor directionality [1, 20].

Chemical signals used in agonistic interactions are most commonly related to social status organization, territoriality or defense [21]. Volatile pheromones are known to signalize dominance status or resource ownership information in diverse taxa, from lobsters and crayfish [22, 23], cockroaches [24], fish [25, 26], salamanders [27] and lizards [28], to various mammals (e.g. [29, 30, 31, 32]). Contrary to these relatively static roles, the function of volatiles as temporally dynamic signals has been reported only rarely. A part of the reason may lie in the difficulty to trace these emissions in real-time during the contest behavior (after [33]). So far, such data have been obtained through visualization or direct quantification of urine release containing volatile signals in aquatic animals (including decapods and fish [34, 35, 36]), or by continuously sampling the airspace around the contestants and supplying it directly to the analytical instrument, which is possible only in relatively small organisms [33, 37]. Although all these studies showed an increase of volatile amounts in the aggressive context of the interaction, only urinary signals could be linked to offensive behavior of the emitters and categorized as aggressive. Olfactory signals of hymenoptera in the studies of Gobault and coworkers [33, 37], on the other hand, were shown to represent a weapon of rearguard action by the loser, thus providing another example of chemical defense that is particularly common among arthropods [38, 39].

**Fig 1:**
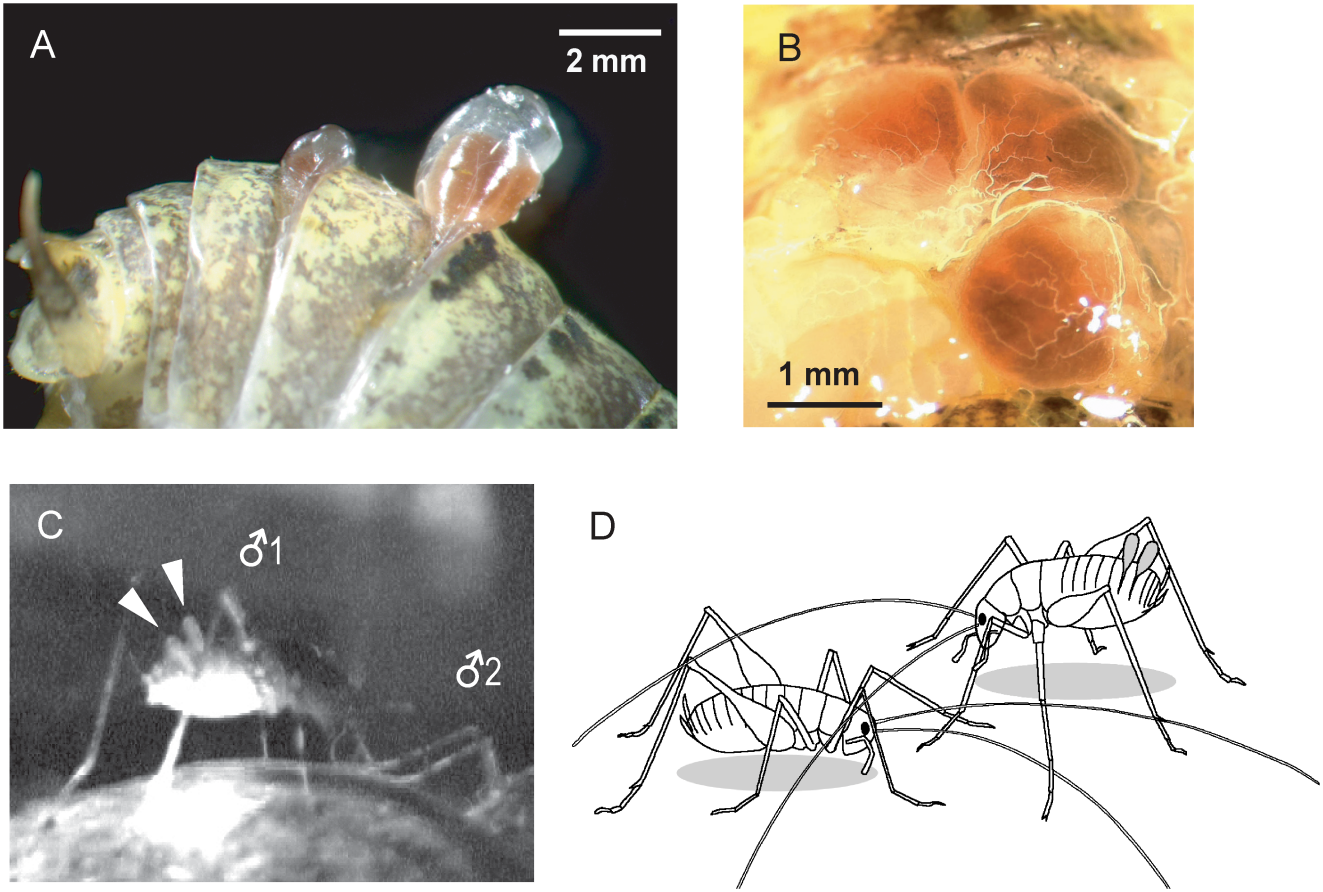
Scent glands in *T*. *neglectus* and their exposure to abdominal surface. (A) Protrusion of dermal bulbs containing gland sacks in the intersegmental regions between 5^th^−6^th^ and 6^th^−7^th^ abdominal segments, induced by a slight compression of the male’s abdomen. (B) Morphology of glands sacks, exposed by removing the respective abdominal tergites (above is anterior). One of the sacks was emptied by puncturing to demonstrate its liquid content. (C) Photo of a male protruding both gland bulbs (arrows) during interaction with another male. Note the aggressive body posture of the focal male. (D) Drawing reconstruction of a male pair during an encounter. The dominant male in the aggressive posture (right) protrudes both gland bulbs.

Here we describe a unique example of temporally modulated olfactory threats among terrestrial animals; signals that are emitted via protrusive, male-specific scent organs in the cave cricket *Troglophilus neglectus* (Orthoptera, Rhaphidophoridae). This nocturnal troglophilic insect, inhabiting karstic regions of the northern Balkans [40, 41], possesses two pairs of large hypodermal gland sacks dorsally in the abdomen [42]. In sexually mature males, these sacks are filled with a red, strongly aromatic secretion, and can be transiently exposed to the body surface [42, 43]. They protrude either as one or both pairs, each within an individual dermal bulb between the subsequent body segments (Fig 1). Since protrusion of these bulbs is clearly visible, it can be quantified and related to behavior of both the signaler and the receiver(s); in that way, the primary function of the emitted odor was recently linked to male agonistic behavior [43]. However, no details of male contests, their relation to the occurrence of gland tissue protrusion or the correlation of such signaling by each individual with the contest outcome has been given to suggest some more specific function of the odor.

We present data on the structure of dyadic contests between physically matched individuals, and correlate the levels of behaviorally expressed aggression and dominance status of the individuals with the measure on the frequency and intensity of their gland tissue protrusion, presumably associated with the amount of released odor. To provide basic support for this association, we monitored the presence of odor during and in the absence of gland protrusion. By preventing gland protrusion, we directly tested the influence of the released odor on the receiver’s behavior. To our knowledge, our data provide the first evidence on olfactory signals mediating information on aggressive intent in a terrestrial species, which is achieved via temporally modified emission of odor along the changing behavioral context.

## Materials and methods

### Animal collection and rearing

The animals were brought into the laboratory at the end of June as last larval instars collected from a wild population in a cave close to Most na Soči, NW Slovenia. Males were reared individually in plastic containers (9 x 9 x 18 cm) furnished with moistened turf and bark, under 16h/8h light/dark photocycle (reversed), at 19 − 20°C and room humidity kept above 60%. The occurrence of the final molt in males was checked and recorded daily. Animals were fed fish food and were provided with water ad libitum.

### Behavioral set-up

*T*. *neglectus* is a nocturnal species, so the experiments were conducted during the scotophase, using red illumination. The room temperature was 21 − 23°C and the humidity between 55 − 65%. A glass container (24 x 38 x 24 cm / w x l x h) filled with a layer of moistened turf, which was covered by a black cotton sheet, served as the experimental arena. Instead of placing the cotton directly on the glass, we used turf as a proxy of a natural substrate, to enable effective transmission of vibration cues produced by the animal movements. After each experimental trial, the cotton sheet was replaced and the terrarium walls were cleaned with acetone to remove contact semoichemicals from the arena, while the room was well ventilated to remove the olfactory residuals. Every five trials, turf was replaced and the complete terrarium was cleaned with acetone. Light was provided by two 11W table lamps covered with red cellophane foil, resulting in light transmittance (λ > 610 nm) undetectable by this species (with normally developed and functional eyes [44]). The black substrate sheet and additional external black panels kept background light reflections low. Recording was conducted by a Canon camcorder (type XM2) positioned at the long side of the arena at ca. 30° above its horizontal plane. By continuously focusing on the encountering animals, such records enabled a clear detection of the presence and intensity of gland tissue protrusion (see also below). In the post-encounter periods, additional observer’s comments were provided that reflected the state of protrusion in any individual outside the recording focus.

### Behavioral experiments

To eliminate the influence of physical characteristics on the fight intensity and outcome, we staged dyadic contests between males matched in size (with the left hind leg femur length differing up to 5%) and body mass (with the weight differing up to 5%; after [6]). At the time of the experiments, the males were between 14 − 53 days old, and the age difference between the paired individuals was ≤ 13 days. This age difference represented the main source of their potential asymmetry relating to aggressive motivation, which may be considered to increase with aging because of progressively decreasing mating opportunities [45].

We carried out four experimental treatments using the same set of individuals: the control treatment with intact individuals, and three additional treatments, in which protrusion of gland tissue was prevented in both contestants simultaneously (one symmetric treatment) and in each one of the contestants separately (two asymmetric treatments) to manipulate signal exchange.

The abdominal cuticle over the glandular region was covered with a few layers of correction paint (non-toxic), which completely prevented gland tissue protrusion during the experiment, but could be easily removed thereafter. To limit the effect of previous experience, males were kept separated for at least 24h before reuse (following the manipulative experiments in crickets [46]), which was supported by the absence of any long-term dominance effects observed in our previous study [43] and in preliminary experiments of the current one. In addition, treatments following the control trials were carried out in a randomized order.

The opponents were individually marked at least half an hour prior to the control experiment. They were carefully transferred to the opposite sides of the arena; the start of an experimental trail was considered their first encounter, and its finish 15 minutes thereafter. In total we analyzed 25 male-male control trials including 50 individuals (exhibiting 7–27 encounters per trial), and 15–17 trials in each of the further treatments including 30–34 individuals (exhibiting 7–40 encounters per trial). Only those pairs were considered in the analysis that exhibited agonistic behavior above the threshold, set to at least 7 mutual encounters in the 15-minute period, and aggression indexes of both opponents being at least 1 (see Data analysis). Excluded were also the trials including a combination of courtship and agonistic behavior (often detected in this species in the absence of receptive females [43]). In the evaluation of manipulation effects only those pairs were considered that showed agonistic behavior above the threshold criteria in at least two additional treatments beyond the control.

### Testing the presence of volatiles

The main volatile compound of gland secretion from *T*. *neglectus*, 5-Methyl-2-phenyl-2-hexenal, has been identified previously from whole-gland extraction by a solvent [47]. Here, we tested for the presence of volatiles emitted from protruded glands in an intact animal, focusing on short time periods typical for male contests. We conducted volatile collection at the constant temperature of 21°C (+/-0.5°) and humidity between 60 − 65%. We induced protrusion of either one or both gland bulbs by a slight lateral compression of the abdomen; in the former case, the posterior-next intersegmental region was glued by wax. The exposed tissue was immediately introduced into a 5-ml glass vial through a ca. 3–4-mm opening made at its bottom (surrounded by Teflon tape for sealing) for various time periods between 5 − 20 seconds, while pressing the surrounding cuticle firmly against the Teflon. After incubation, the vial opening was quickly closed with aluminum foil and plugged, and its content was sampled by SPME for 30 minutes (polydimethylsiloxane coated fiber, 100μm, Supelco, Bellefonte, PA, USA). To check for the putative presence of volatiles in the absence of gland protrusion, we kept individual males in 50 ml glass jars for 12 − 18 hours isolated from any conspecific stimuli, while sampling the headspace continuously with SPME. The SPME fibers were analyzed by gas chromatography-mass spectrometry (GC-MS; splitless mode) using an Agilent 6890N GC (Agilent, Santa Clara, CA, USA) coupled to a 126 5973 mass selective detector. GC was fitted with a DB-23 column (60 m x 0.25 mm diameter, 127 0.15 μm film thickness; J&W Scientific, Folsom CA, USA) and programmed from 40°C for 1 min, 10°C min^-1^ to 230°C, using helium carrier gas. SPME fibers were desorbed in the injector port for 1 min.

### Behavioral analysis

Video records were analyzed using Microsoft Windows Movie Maker 2.1. The start of an encounter was considered the time of the first contact between the rivals, and its end as the time of the loser’s retreat, which was not followed by a subsequent frontal contact with the opponent for at least 5 seconds. In the interactions with mutual escalation of aggression to the level 2 or 3 (see Table 1), encounter duration was measured with 0.5 seconds resolution, and calculated were trial means.

**Table 1:** Types of behaviors displayed during agonistic interactions of *T*. *neglectus* males with intensity scores.

Behavior (encounter)	Level of escalation	Intensity score	Behavior (post-encounter)	Intensity score	Total score
Transient contact	0	0	avoid / flee	0	0
			stay	1	1
Antennation	I	1	avoid / flee	0	1
			stay	1	2
			chase	2	3
Aggressive posture	II	2	avoid / flee	0	2
			stay / circulate	1	3
			chase	2	4
Attack (fight)	III	3	avoid / flee	0	3
			stay / circulate	1	4
			chase	2	5

Note that behavior expressed at a particular level of aggressive escalation includes all the previous level elements. Fighting, for example, also includes intense antennation and the aggressive posture display of the opponents.

For each encounter, we identified the individual that initiated it and ascribed to it an intensity score reflecting the level of aggressive escalation expressed before (0–3), and immediately after, the encounter resolution (0–2; Table 1). For each individual, we calculated three behavioral parameters: the mean level of aggressive escalation (i.e., “aggression level”), the “aggression index” and the “dominance index”. The aggression level equals the mean (trial) intensity score ascribed to behavior prior to encounter resolution, which was used in most evaluations. The aggression index represents the mean (trial) intensity score calculated from the complete behavioral performance, during and after encounters (Table 1), which was used only in correlation with the overall degree of gland protrusion within a trial. The reasoning behind such scoring is that the level of aggressive motivation is likely also to have influenced post-encounter behavior (e.g., a highly aggressive winner would chase the opponent after its retreat, while a less aggressive winner would not), and that such a total measure of aggression would better correlate with gland protrusion, if the latter is to mediate the animal’s intent. The “dominance index” was calculated as the proportion between a male’s aggression index and that of its opponent, by which the higher index defined a dominant male. This male was in most cases also the one winning most encounters; using this index enabled us to identify a male as dominant even in cases when both opponents won the same number of encounters.

### Quantification of gland tissue protrusion

A part of our data analysis (and interpretation) is based on two assumptions: the amount of the emitted odor is proportional to the frequency and extent of gland tissue protrusion, and its protrusion through individual dermal bulbs has a simple additive effect (see also Discussion). In line with these assumptions, we evaluated the extent of gland bulb protrusion for each individual in each encounter and post-encounter periods, scoring it from 0–2 (reflecting its natural occurrence): 0 − no protrusion, 0.5 − slight protrusion of the anterior bulb, 1 − the anterior gland bulb protruded, 2 − both gland bulbs protruded. For each male, we calculated the overall (trial) means for the different contexts (encounter, post-encounter), as well as the means for each level of aggression expressed by both winners and losers of individual encounters. These means were then normalized, resulting in a combined measure of the relative protrusion frequency and extent, referred to as the protrusion “degree” throughout the manuscript.

### Statistical analysis

We conducted the statistical analysis and designed the charts using SPSS 14.0. (SPSS, Inc., Chicago, Illinois, USA). For distributions of behavioral data, which were mostly non-parametric, we calculated the median and interquartile ranges and tested for significant differences between them by the Wilcoxon signed-ranks test and the Mann-Whitney U test for the paired and unpaired data, respectively. We tested for data correlation by the Spearman rank correlation. We used Adobe Photoshop 8.0. and Adobe Illustrator 11.0 (Adobe Systems, San Jose, CA, USA) to make the drawing and for final representation of charts.

## Results

### General characteristics of agonistic behavior in *T*. *neglectus*

In mutual encounters, males displayed stereotyped agonistic behavior that could be ascribed the following levels of aggressive escalation: level 0 –transient contact; level 1 –high frequency antennal fencing (antennation); level 2 –elevated position of the body and particularly the abdomen (i.e., aggressive posture; Fig 1C and 1D); and, level 3 –foreleg kicking and / or thrusting into the rival (attack / fight when mutual; Table 1). Following the conclusion of an encounter, the winner either shortly retained the position, sometimes making a local circular “parade”, or chased the loser for some distance. Except for a few initial encounters, behavior was expressed at a particular level of aggressive escalation directly, without proceeding gradually though the lower levels, and instantaneously including all the previous level elements. The encounters were generally brief; their average duration in the case of mutually escalated aggression ranged around 2.75 seconds (median, Q1 − Q3 2.0–3.55, N = 14).

Only 20% of the encounters in the control treatment (N = 362, encounters with a clear resolution) were resolved whenever males displayed behavior at the same aggression level; in that case level 2 was expressed most commonly (41%, N = 148). In other cases, the display of the winner was more aggressive than that of the loser. The aggression level of dominant males ranged around 1.6 (median, Q1 − Q3 1.3–1.9, N = 25) and that of subordinate males around 0.9 (median, Q1 − Q3 0.5–1.12, N = 25), with their aggression indexes ranging around 2.55 (median, Q1 − Q3 2.0–3.29, N = 25) and 1.17 (median, Q1 − Q3 0.5–1.33, N = 25), respectively. The dominants, which in 81% of the contests were older than their opponents (N = 21, the pairs with age difference), initiated significantly more encounters than subordinates (median 84%, Q1 − Q3 75–93%, U = 29, N_1_ = N_2_ = 25, Mann-Whitney U test, P < 0.001).

### The presence and composition of volatiles

**Fig 2:**
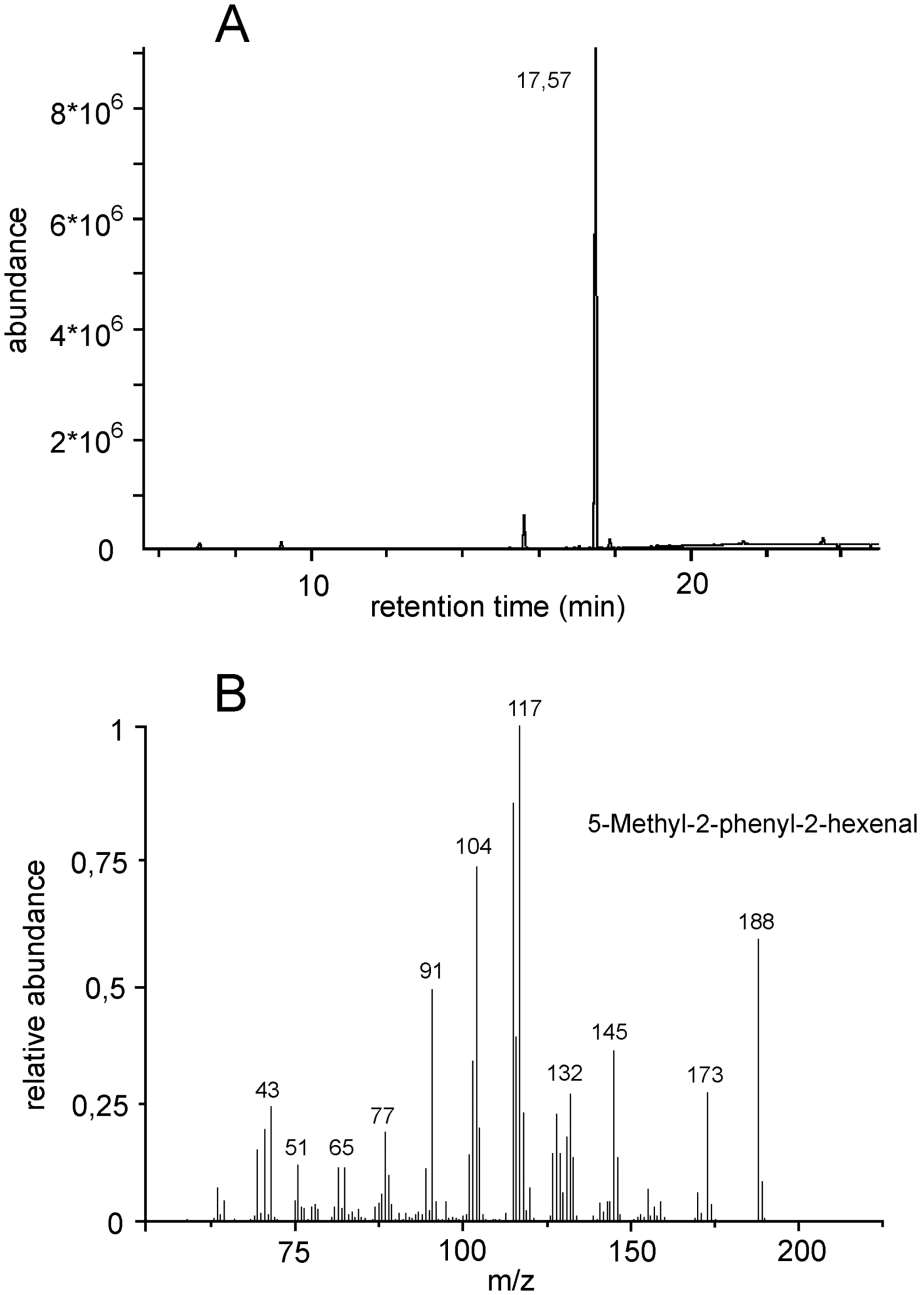
The volatile extract analysis from scent glands of *T*. *neglectus*. (A) Gas chromatogram of the SPME equilibrium extraction of the sample obtained by 10 seconds incubation of both gland bulbs. (B) Mass spectrum of the major compound, 5-Methyl-2-phenyl-2-hexenal.

Gas-chromatographic analysis of SPME extracts confirmed the presence of 5-Methyl-2-phenyl-2-hexenal, eluting at around 17.55 min, as the major and largely prevailing volatile compound in emission from protruded scent glands in *T*. *neglectus* (Fig 2). An additional minor, unidentified, compound of a very similar structure (most likely the derivate of the major compound; J. Millar, personal communication), eluted about 2 min sooner in all samples. No other volatile compounds were found to be consistently present. Despite the short incubation of the protruded gland tissue (5 − 20 s), large amounts of the major compound were indicated by the analysis of SPME equilibrium extracts (Fig 2A). At the same time, no trace of this compound was revealed in the samples from males incubated for long periods with glands retracted. Our collection method, however, was apparently too crude (and the SPME extraction also not optimal for quantification; J. Millar, personal comm.) to show the correlation between the relative amounts of odor and the different periods and extent of protrusion tested.

### Gland protrusion—Context, aggression and dominance

Gland protrusion was expressed in two different behavioral contexts: during the male-male encounters in relation to direct contacts, and in the post-encounter periods in the absence of contacts. Clearly, the primary trigger of gland protrusion within each trial was the contact between the opponents. Direct contacts represented the predominating context of gland protrusion both with respect to proportion of males that signaled in such a way (96%, N = 50 males), and the total number of encounters in the control treatment; in 82% of encounters a dominant male was protruding the glands during contacts, and in 53% of encounters a subordinate male was protruding them while contacting the opponent (N = 448 encounters). Typically, glands protruded instantaneously at the first contact in an encounter, and were present throughout the interaction. In most cases they were retracted as soon as the opponents separated.

Gland protrusion following the encounters was expressed less often, occurring in 48% of the males (N = 50) following 28% and 4% of encounters by dominant and subordinate males, respectively (N = 448). Such protrusion was mostly a discrete event, occurring shortly after the encounter resolution, although it could also represent a continuation from an encounter, typically in association with high aggression levels. While protruding glands after an encounter, males either stayed in one place (often grooming) or moved around the arena (often chasing or following the opponent). Duration of protrusion after the encounters apparently increased with the increasing level of the signaler’s aggression (not quantified).

**Fig 3:**
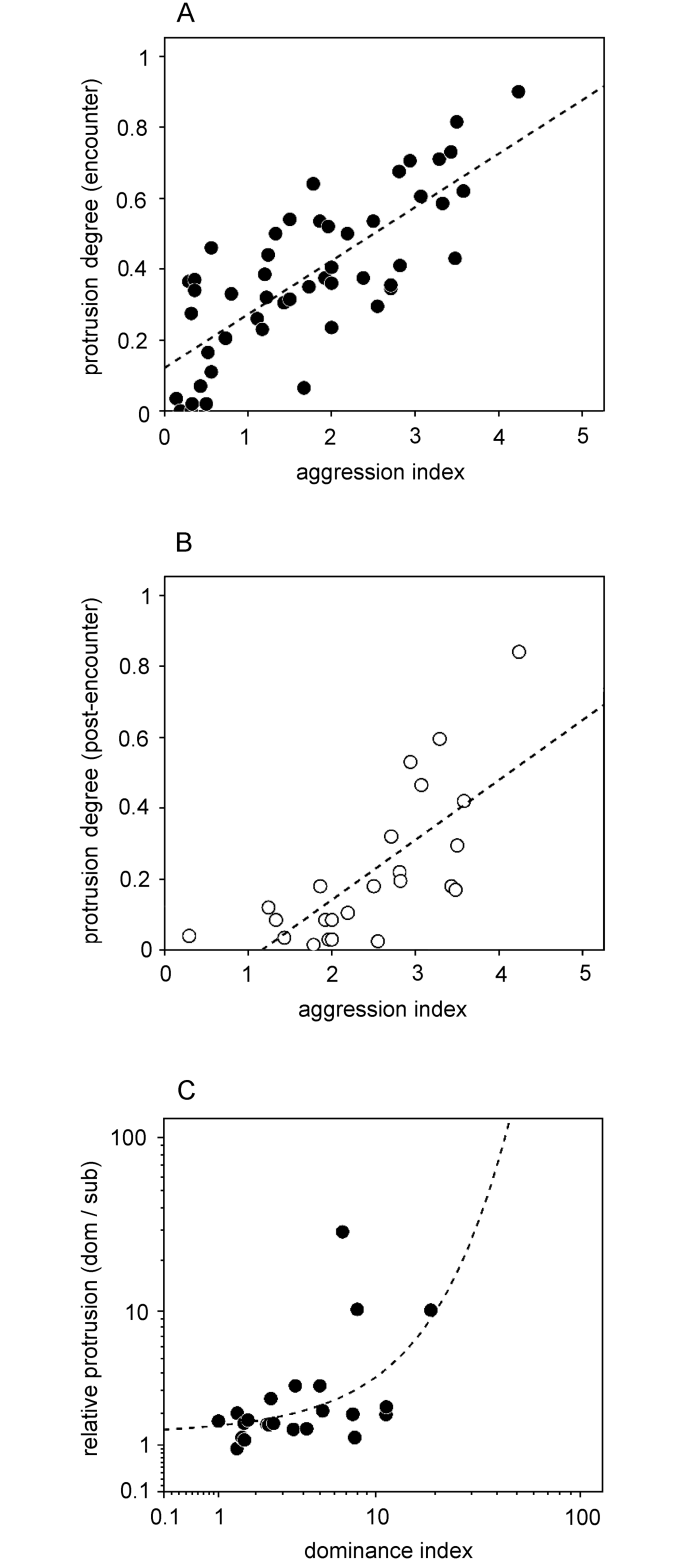
Correlation between gland protrusion, aggression and dominance. Shown is the protrusion degree of individual males for the (A) encounter and (B) post-encounter periods (N = 48 and N = 24, respectively) in the control treatment with respect to their agression index, and (C) the proportion of the protrusion degree between dominant and subordinate males during the encounters with respect to their dominance index (N = 23). Added are regression lines of best fit (A, B—linear: R^2^ = 0.594 and R^2^ = 0.533, respectively; C—exponential: R^2^ = 0.273).

In both behavioral contexts, the degree of gland protrusion linearly increased with the increasing intensity of the signaler’s aggression (i.e., aggression index; Fig 3A and 3B; Spearman rank correlation: r_s_ = 0.741, N = 50, P < 0.001, and r_s_ = 0.716, N = 50, P < 0.001, respectively). The relative protrusion degree with respect to the rival’s was strongly positively correlated with the dominance index of the individuals (Fig 3C; Spearman’s Correlation, r_s_ = 0.891, N = 23, P = 0.009); i.e., the more dominant a male was, the more frequently and intensely he protruded the glands compared to his rival.

**Fig 4:**
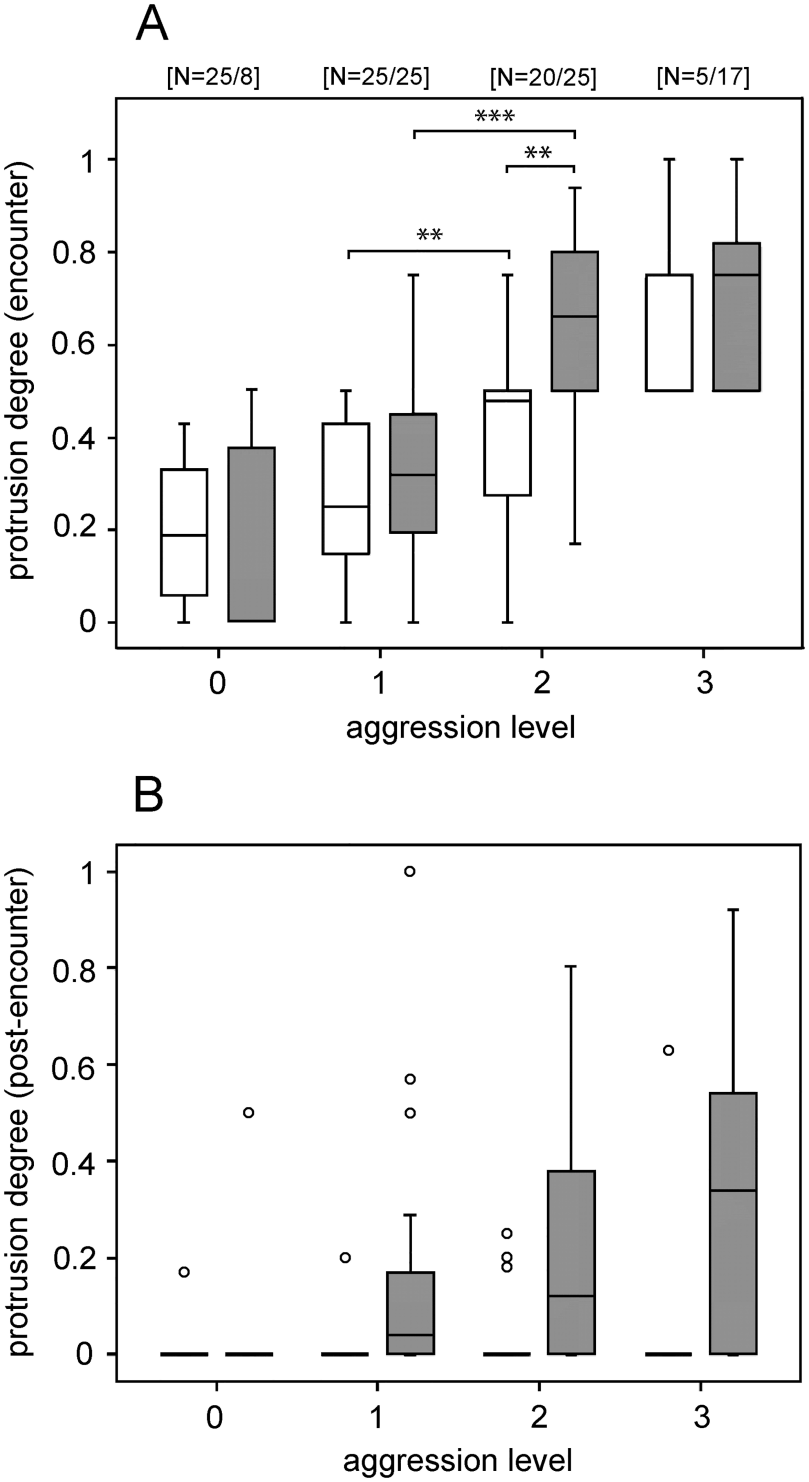
Gland protrusion at individual levels of aggressive escalation. Shown is the protrusion degree for (A) the encounter and (B) the post-encounter periods expressed by winners (grey) and losers of individual encounters (white / left position) at individual aggression levels in the control treatment. Data are shown as box-plots with median and interquartile ranges; circles represent outliers. Shown are the number of trials with wins and losses for each level (N_lose_/N_win_; valid for A and B), and significant differences are indicated (*** P < 0.001, ** P < 0.01; Wilcoxon signed-ranks and Mann-Whitney U test, for between and within level comparisons, respectively).

During the encounters, gland protrusion degree statistically significantly increased only between the first and the second level of aggressive escalation, and did so in both winners and losers of individual encounters (Wilcoxon signed-ranks test, T = 1, N = 24, P < 0.001 and T = 10.5, N = 20, P = 0.002, respectively; Fig 4A). The difference in the protrusion degree between winners and losers was statistically significant only at the aggressive level 2 (i.e., aggressive posture; Mann-Whitney U test, N_1_ = 16, N_2_ = 12, P = 0.004; Fig 4A). After the encounters, glands were protruded almost exclusively by the encounter winners, with the degree of protrusion increasing more steadily with the winners’ aggression (Fig 4B).

### Signal exchange manipulation

**Fig 5:**
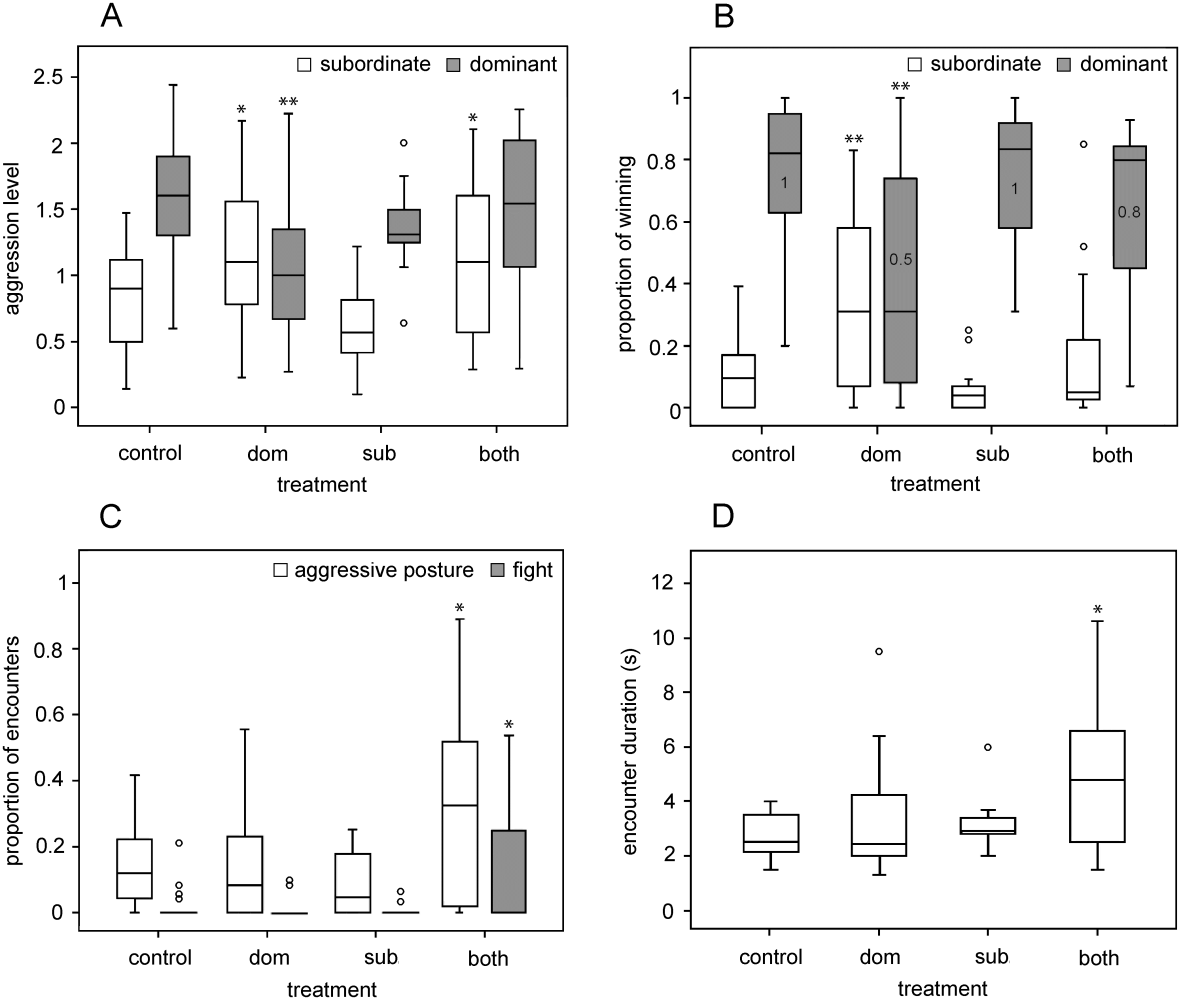
Effects of the manipulative treatments. (A) The level of aggressive escalation, (B) the proportion of encounters won by the males defined as dominant and subordinate in control trials (grey and white boxes, respectively; in (B) the values in boxes show the proportion of dominant males in the respective treatments), (C) the proportion of encounters with aggression mutually escalated to the same level (white boxes: aggressive posture, grey boxes / right position: fight), and (D) duration of these mutually aggressive interactions in different treatments. The treatments consist of the control with intact individuals (control), and of treatments with gland protrusion prevented, i.e., in the males defined as dominant in the control (dom), the males defined as subordinate in the control (sub) and in both males in a pair (both). Data are shown as box-plots with median and interquartile ranges, circles represent outliers. Asterisks show the degree of significant differences between the control and the indicated data sets (* P < / = 0.05, ** P < 0.01; Wilcoxon signed-ranks test). N = 15 − 17 (for A, B) and 5–17 (for C, D).

Preventing gland protrusion largely influenced the measures of aggression expressed by both treated and untreated individuals, as well as the outcome and duration of their encounters, compared to control trials (Fig 5). After gland protrusion was prevented in males defined as dominant in control trials, their level of aggression highly significantly decreased (Wilcoxon signed-ranks test; T = 17, N = 17, P = 0.005), while that of their opponents, defined as subordinate in control trials, significantly increased (Wilcoxon signed-ranks test; T = 35.5, N = 17, P = 0.05; Fig 5A). Consequently, the proportion of winning encounters changed highly significantly, decreasing for dominants and increasing for subordinates (Wilcoxon signed-ranks test; T = 1, N = 15, P = 0.001 and T = 8.5, N = 15, P = 0.006, respectively), resulting in close matches in dominance between opponents (Fig 5B). After gland protrusion was prevented in the subordinate males, their aggression level markedly, but non-significantly, decreased (Wilcoxon signed-ranks test, T = 26, N = 15, P = 0.096; Fig 5A), as did the share of encounters they won (Wilcoxon signed-ranks test, T = 14, N = 15, P = 0.091; Fig 5B). At the same time, the variation in aggression in both groups of males markedly narrowed compared to control trials (Fig 5A).

When gland protrusion was prevented in both opponents, the aggression level of subordinate males significantly increased (Wilcoxon signed-ranks test, T = 21, N = 15, P = 0.048; Fig 5A) and therewith also the proportion and duration of encounters mutually escalated in aggression (Fig 5C and 5D). There was a significant increase in the proportion of encounters that included the aggressive posture display of both opponents (level 2; Wilcoxon signed-ranks test, T = 17, N = 15, P = 0.026), resulting mostly from the increase in the frequency of fights compared to the control (level 3; Wilcoxon signed-ranks test, T = 1, N = 15, P = 0.017; Fig 5C). Although such maximal contest escalation was absent in the majority of control trials, as well as in those including asymmetric treatments (Q3 = 0, maximum = 0.06–0.21), it was expressed in 50% of the trials with symmetric treatments of the opponents (Q3 = 0.25, maximum = 0.54). These escalated interactions lasted on average up to 4 seconds in the control trials (median 2.75, Q1 − Q3 2.06–3.55; Fig 5D), while their average duration increased significantly up to 10.6 seconds when gland protrusion was prevented in both opponents (median 4.3, Q1 − Q3 2.72–6.9; Wilcoxon signed-ranks test, T = 4.5, N = 10, P = 0.019). At the same time, the proportion of winning by dominant males only slightly (non-significantly) decreased (T = 38.5, N = 15, P = 0.222), and they retained dominance in 80% of the trials (Fig 5B).

## Discussion

### Information content of olfactory signals in *T*. *neglectus*

We have demonstrated that 5-Methyl-2-phenyl-2-hexenal (5,2,2-MPH), previously identified from gland secretion in *T*. *neglectus* [47], represents the major volatile compound also in the emission from protruded glands and therewith the pheromone involved in agonistic behavior of the species. This substance has been found in different plants and plant-derived food [47, 48, 49], so its production in *T*. *neglectus* is most likely based on nutrition. In the absence of other volatile compounds (except of the likely derivate of 5,2,2-MPH), such a pheromone system has no obvious potential to provide information on the signaler’s status or aggression in its structure. Chemical signals in glandular or urine excretions may generally reveal such information directly via the presence of metabolic products of neurotransmitters or neurohormones that control for aggressive behavior [50, 51], or may encode information on the signaler’s experience or identity in the composition or ratio of different volatile compounds [24, 52]. Since the pheromone system of *T*. *neglectus* meets neither of these options, the males appear to signal all information via the context and quantity of the odor emission.

We presumed that the quantity of the emitted odor in *T*. *neglectus* is correlated with the frequency and extent of gland tissue protrusion, and that protrusion of individual dermal bulbs emits similar odor amounts. This assumption is supported by the general fact that the diffusion rate is proportional to the diffusion surface area, and by our observations that the two pairs of gland sacks, which may be exposed individually in *T*. *neglectus*, are of similar morphology and typically contain similar secretion amounts. We demonstrated that the odor is emitted exclusively during gland protrusion and in large amounts during the short periods of contests. Although we were not able to provide more details on the protrusion-emission relationship, our study—and particularly the manipulative experiments—provide definite evidence of communication and its effect mediated by the emitted odor.

### Signaling intention to attack

During contests of *T*. *neglectus*, the degree of gland tissue protrusion (and, thus, the amount of the emitted odor) increased along the increasing intensity of the signaler’s aggression. This suggests that the released olfactory signals meet the primary, “context” criterion that defines an aggressive threat [53]. If a signal functions as a threat, it should also reliably predict the imminence of, or escalation toward, the attack (the “predictive” criterion [53]). This predictive criterion, however, is difficult to test, since the effective threats would intimidate the receiver and the signaler’s escalation would not occur [1, 54]. Demonstration of the signal’s predictive value therefore requires an analysis of behavioral sequences considering the sender’s behavior of interest, the receiver’s response and the sender’s subsequent behavior (e.g. [35, 55]). Such an analysis was not applicable to *T*. *neglectus*, since their contests are very short and are expressed in phase rather than in clear succession of behaviors. Still, our data provide clear evidence on the intention-to-attack function of the odor emitted in the course of contests.

Along the increasing levels of aggressive escalation, the degree of gland protrusion increased most strongly in association with the occurrence of the elevated (i.e., aggressive) body posture. This frequent display of body size [1, 56] in *T*. *neglectus* directly precedes and constitutes the attack. It may be thus considered also a threat, since threat signals frequently occur as postures representing the initial position of the fighting technique in a given species [15]. Due to tight coupling of this posture to gland protrusion, a threat function might also be presumed for the released odor. Indeed, only with the mutual aggressive posture display that occurs just prior to a fight, but not at the lower levels of aggressive escalation, was the degree of gland protrusion significantly higher in winners than in losers of individual encounters. This indicates the decisive role of the emitted olfactory signals in conflict resolution particularly in the situation just prior to maximal conflict escalation; in other words, these signals appear to function as an effective threat. This function was directly confirmed in the manipulative experiments, in which the frequency of fights significantly increased after gland protrusion was prevented in both opponents. The extent of this effect was shown after the olfactory signaling was prevented in the dominant males, which subsequently kept their dominance only in 50% of the trials, while losing even a higher share of individual encounters compared to the control treatment. This finding demonstrates that in *T*. *neglectus* the tactile information alone is at best half as effective in convincing the opponent of the signaler’s superiority as it is in combination with olfactory signals. Similar findings have been obtained in the crayfish, *Astacus leptodactylus*, where chemical signals in the urine directed at the opponent proved to be even more important for resolving a contest than other offensive signals and displays [35].

Both crayfish and cave crickets are nocturnal animals and, in the absence of visual information, their chemical signals appear to have a major influence on the contest resolution. To increase detectability of these stimuli, chemical signals of crayfish are directed towards the opponent with the gill current [35]. Cave crickets, on the other hand, possess extremely long antennae, which during frontal apposition of the contestants reach completely over the opponent’s body and, thus, may detect the opponent’s olfactory signals adjacent to the source (see Fig 1D).

To our knowledge, there are no other examples of olfactory signals with a threat function demonstrated for terrestrial animals. Such a function may be possible for excretions of pre-orbital glands in Cervoidea, such as deer [57], musk deer [58] and gazelles ([59], cited from [60]), which open or rub these glands during fighting and/or threatening. It is yet unknown, whether and in what extent the emitted odor influences contest resolution in these species.

### Signaling dominance

During the contests in *T*. *neglectus*, gland protrusion was expressed to some degree by both winners and losers, while after their resolution it was displayed almost exclusively by winners. It is very likely that the odor emitted in this context is advertising dominance, and particularly victory (see [1], for a functional classification of aggressive signals), since victory display is defined as post-contest signaling performed by the winner and not the loser of a contest [61]. Such a function has been proposed, for example, for aggressive stridulation after fighting bouts in crickets, which is displayed either exclusively or at much higher rates by winners compared to losers [62, 63, 64, 65]. Two possible functions were generally proposed for a victory display: it may discourage the loser to initiate any further fights, and/or advertise information on the winners’ good condition to putative third party receivers [61, 66]. In the light of the latter possibility, we may now understand also the previously unexplained function of gland protrusion by *T*. *neglectus* males in the presence of females, which didn’t influence female attraction nor receptivity [43]. These signals may have represented an advertisement of the male’s motivation to fight for a perceived mating opportunity, and may be classified as ownership signals or mild long-distance threats [1].

### Decreasing the costs of conflict

Engaging in contests may be costly in terms of energy expenditure, injuries, or an increased risk of predation [3, 67] and communication functions to reduce these costs [1, 68]. The costs of conflict were not in the focus of our research, however, results of the manipulative experiments provided evidence on the above assumption, with the contest duration and the frequency of fights serving as the indicators of conflict-related costs (see [65, 69]). We found both contest parameters to be significantly increased after olfactory signaling was prevented in both opponents, while none of the asymmetric treatments influenced their values. These data are similar to findings in crickets, in which only symmetric handicaps, including disabled mandibles, maxillae, blinding of crickets [46] or prevention of stridulation [65], caused a significant increase in duration and / or aggression of male-male interactions. In line with these data, our study provides further evidence that signaling decreases the costs of aggressive interactions, and shows its efficiency as far as one of the contestants remains intact.

### Agonistic signaling modes of Orthoptera

The use of olfactory signals in agonistic contexts, such as expressed in *T*. *neglectus*, is an exception among Orthoptera, which settle contests mostly by means of acoustic and visual signaling. In crickets, katydids and weta, information on male size and putatively their aggressiveness and post-contest status is communicated by sound or substrate vibration signals (e.g. [63, 70, 71]). Males of many of these species possess enlarged mouthparts used for visual assessment of strength in the non-contact phase and for providing physical damage in the final phase of conflict escalation [6, 72, 73, 74]. Most of these signaling modes are unavailable for cave crickets, which are devoid of sound production [75] and mainly inhabit subterranean places or are nocturnal [40, 76]. Accordingly, scent organs that might be used for communication developed repeatedly in Rhaphidophoridae (see [43]), but were functionally investigated only in *T*. *neglectus*. Our study shows that volatile signals in males of this species represent a close functional correlate of aggressive stridulation in crickets, which has been proposed to function as a mild aggressive threat during contests [1, 62] and signalizes dominance thereafter. Such use of olfactory signals appears to be unique not only among the Orthoptera, but among all previously studied agonistic systems of terrestrial animals.
